# P2000 - A high-nitrogen austenitic steel for application in bone surgery

**DOI:** 10.1371/journal.pone.0214384

**Published:** 2019-03-26

**Authors:** Mustafa Becerikli, Henriette Jaurich, Christoph Wallner, Johannes Maximilian Wagner, Mehran Dadras, Birger Jettkant, Fabian Pöhl, Merlin Seifert, Ole Jung, Bojan Mitevski, Ahmet Karkar, Marcus Lehnhardt, Alfons Fischer, Max Daniel Kauther, Björn Behr

**Affiliations:** 1 Department of Plastic and Reconstructive Surgery, BG University Hospital Bergmannsheil, Ruhr-University Bochum, Bochum, Germany; 2 Department of General and Trauma Surgery, BG University Hospital Bergmannsheil, Ruhr-University Bochum, Bochum, Germany; 3 Chair of Materials Technology, Ruhr-University Bochum, Bochum, Germany; 4 Department of Materials Science and Engineering, University of Duisburg-Essen, Duisburg, Germany; 5 Departmen of Orthopaedics and Trauma Surgery, University Hospital Essen, University of Duisburg-Essen, Essen, Germany; Institute of Materials Science, GERMANY

## Abstract

Optimal treatment of bone fractures with minimal complications requires implant alloys that combine high strength with high ductility. Today, TiAl6V4 titanium and 316L steel are the most applied alloys in bone surgery, whereas both share advantages and disadvantages. The nickel-free, high-nitrogen austenitic steel X13CrMnMoN18-14-3 (1.4452, brand name: P2000) exhibits high strength in combination with superior ductility. In order to compare suitable alloys for bone implants, we investigated titanium, 316L steel, CoCrMo and P2000 for their biocompatibility and hemocompatibility (according to DIN ISO 10993–5 and 10993–4), cell metabolism, mineralization of osteoblasts, electrochemical and mechanical properties. P2000 exhibited good biocompatibility of fibroblasts and osteoblasts without impairment in vitality or changing of cell morphology. Furthermore, investigation of the osteoblasts function by ALP activity and protein levels of the key transcription factor RUNX2 revealed 2x increased ALP activity and more than 4x increased RUNX2 protein levels for P2000 compared to titanium or 316 steel, respectively. Additionally, analyses of osteoblast biomineralization by Alizarin Red S staining exhibited more than 6x increased significant mineralization of osteoblasts grown on P2000 as compared to titanium. Further, P2000 showed no hemolytic effect and no significant influence on hemocompatibility. Nanoindentation hardness tests of Titanium and 316L specimens exposed an indentation hardness (H_IT_) of about 4 GPa, whereas CoCrMo and P2000 revealed H_IT_ of 7.5 and 5.6 GPa, respectively. Moreover, an improved corrosion resistance of P2000 compared to 316L steel was observed. In summary, we could demonstrate that the nickel-free high-nitrogen steel P2000 appears to be a promising alternative candidate for applications in bone surgery. As to nearly all aspects like biocompatibility and hemocompatibility, cell metabolism, mineralization of osteoblasts and mechanical properties, P2000 was similar to or revealed advantages against titanium, 316L or CoCrMo.

## Introduction

For many decades surgical reconstruction plates, nails and screws on the basis of steel and titanium alloys are used for reconstruction of bone defects in orthopedics, trauma-, hand-, craniofacial surgery [1,2]. Both share advantages and disadvantages. Today, high-strength TiAl6V4 (GRADE 5) is used in Europe and in part also the US [3,4] and shows a good biocompatibility as well as corrosion resistance [5,6]. However, titanium alloys reveal an increased risk of cold welding with the impossibility of plate removal during revision interventions or material removal and much higher production costs are necessary as compared to stainless steels [1,7,8,9]. Additionally, more postoperative implant failures were observed after using titanium compared to stainless steel implants [10]. In general, stainless steel alloys, such as the commonly used 316L-type of steels, show good properties as to mechanical strength, toughness as well as cyto- and biocompatibility; thus they are the most frequently used implant materials for internal fixation in orthopedics [1,7,11,12]. A major disadvantage of these steels is that they could release metal ions such as nickel, that have been reported to cause allergic reactions in patients [13,14]. In order to avoid this disadvantage, alternative alloys have been investigated like nickel-free cobalt-chromium-molybdenum alloy (CoCrMo, here: CoCr28Mo6), that circumvents the existing allergic reactions of nickel [15]. Nevertheless, CoCrMo could induce pro-inflammatory cytokines, osteocyte apoptosis and could block the calcium influx in osteoblast-like cells [16,17,18]. The nickel-free, high-nitrogen austenitic steel X13CrMnMoN18-14-3 (1.4452, brand name: P2000) exhibits high strength in combination with superior ductility [19]. Compared to 316L steel, P2000 also provides a better corrosion resistance, which is supported by the high nitrogen content [12].

As we could show in a previous study, with increasing age, bone homeostasis is affected by progressive deprivation of cell function and proliferation, whereas markers related to osteogenesis as well as osteoclastogenesis were significantly decreased in aged individuals [20]. These alterations in bone activity are accompanied with a 10-fold-increased fracture risk in aged compared to young individuals [21,22]. Thus, for optimal treatment of bone fractures with minimal complications, it is necessary to develop apply implant alloys for application in bone surgery that combine high strength with high ductility.

In order to compare suitable alloys for bone implants, the aim of this study was to investigate materials like titanium, 316L steel, CoCrMo and P2000 as to their biocompatibility and hemocompatibility, cell metabolism, mineralization of osteoblasts as well as electrochemical properties.

## Material and methods

### Preparation of the material specimens

Biomedical grades of Titanium (TiAl6V4, GRADE 5), 316L (X2CrNiMo17-12-2, X2CrNiMo19-12, X2CrNiMo18-14-3), low-carbon CoCrMo (CoCr28Mo6)) and P2000 (X13CrMnMoN18-14-3) samples with two different geometries were prepared: a) round samples of 18 mm diameter and 1 mm thickness for almost all experiments and b) rectangular samples of 9,5 mm x 30 mm and 1 mm thickness exclusively for hemocompatibility experiments. After electrolytical polishing, specimens were pickled in 5% HNO_3_ (80°C, 2h), degreased in acetone, sterilized in ethanol and finally washed with distilled water. Samples were kept in a desiccator for protection from humidity. Prior to use, samples were again disinfected with isopropyl alcohol for 5 min and air-dried. Positive controls were performed by growing the cells on plastic and negative controls were performed by cell cultivation on RM-A, a polyurethane film containing 0.1% zinc diethyldithiocarbamate (ZDEC) (Hatano Research Institute, Food and Drug Safety Center, Japan).

### Cell culture

The mouse fibroblast cell line L-929 was purchased from Sigma-Aldrich (St. Louis, Missouri, USA) and was cultured in MEM (Minimum Essential Medium) supplemented with 10% fetal bovine serum (Thermo Fisher Scientific Inc., Waltham, MA, USA), penicillin/streptomycin (100 U/mL each) and L-glutamine in a final concentration of 4 mM. The human osteoblasts were obtained from PromoCell (Heidelberg, Germany) and were grown in osteoblast growth medium (PromoCell, Heidelberg, Germany) without additional supplements. Cells were passaged when they reached about 80% confluence. Mineralization was performed by incubation with osteoblast mineralization medium (PromoCell, Heidelberg, Germany).

### Live-dead staining

Staining of living and dead cells on the surfaces of the specimens was performed by fluorescein diacetate (FDA) and propidium iodide (PI). Material specimens were placed into wells of 12-well plates and were seeded by 2,4x10^5^ cells in 1 ml medium for 24 h. Thereafter, 60 μl PI stock solution (50 μg/ml in PBS) and 500 μl FDA working solution (20 μg/ml in PBS) were added to each well for 3 min at room temperature. Specimens were washed with PBS and results were evaluated microscopically via a Zeiss Axioskop 2 Plus microscope with filters for parallel detection of green and red fluorescence. Pictures were taken using an AxioCam HRc. Results are shown in arbitrary units.

### Cell proliferation

Cell proliferation was measured via the “Cell Proliferation Kit II (XTT)” (Roche Diagnostics, Mannheim, Germany) according to the manufacturer’s instructions. Briefly, material specimens were placed into wells of 12-well plates and were seeded by 6x10^4^ cells in 1 ml medium for 24 h. Cells normally grown on the plastic cell culture plate served as positive controls. Cells grown on RM-A served as negative controls. Thereafter, 500 μl of the XTT labeling mixture was added to the cells for another 4 h. Quantification was performed by measuring the absorbance of 100 μl aliquots in a new 96-well plate by an Elx808 Ultra Microplate Reader (Bio-Tek Instruments GmbH, Bad Friedrichshall, Germany) with filters for 450 and 650 nm (reference wavelength). Results are shown in arbitrary units. Indirect tests were performed by specimen incubation in 1,8 ml medium for 72 h. This conditioned medium was added to 1x10^4^ L929-cells grown for 24 h in wells of 96-well plates. After additional 24 h proliferation was measured according to the manufacturer’s instructions.

### Cytotoxicity

Cytotoxicity was investigated by LDH-Cytotoxicity Assay Kit II (BioVision, Milpitas, CA 95035, USA) according to the manufacturer’s instructions. Cell supernatants from part 2.4. were analyzed.and absorbance was measured like in part 2.4. Results are shown in arbitrary units. Indirect tests were performed by specimen incubation in 1.8 ml medium for 72 h. This conditioned medium was added to 1x10^4^ L929-cells grown for 24 h in wells of 96-well plates. After additional 24 h cytotoxicity was measured according to the manufacturer’s instructions. Controls were also performed as described previously.

### Scanning electron microscope (SEM)

Material specimens were placed into wells of 12-well plates and seeded by 6x10^4^ osteoblast cells in 1 ml medium for 24 h. Cells were fixed in 3% glutaraldehyde at 4°C over night, followed by dehydration using an ethanol series. After sticking onto metal stubs and gold/palladium (60%/40%) coating in a sputter coater (Emitech K500x, Gala Instruments, Bad Schwalbach, Germany), specimens were analyzed by SEM (DSM 962, Zeiss, Oberkochen, Germany).

### Alkaline phosphatase (ALP) activity

ALP activity was investigated by the colorimetric Alkaline Phosphatase Assay kit (abcam, Cambridge, UK) according to the manufacturer’s instructions. Briefly, material specimens were placed into wells of 12-well plates and seeded by 1.25x10^5^ osteoblasts in 1 ml medium for 1 week. Cells grown on the plastic cell culture plate served as positive controls. Absorbance was correlated with the number of harvested cells. Results are shown in arbitrary units.

### RUNX-2 immunofluorescence staining

In order to investigate RUNX-2 protein levels material specimens were placed into wells of 12-well plates and were seeded by 1.25x10^5^ osteoblasts in 1 ml medium for 24 h. Cells grown on the plastic cell culture plate served as positive controls. Medium was changed by osteoblast mineralization medium for additional 48 h. Cells were fixed in 4% paraformaldehyde for 30 min at 37°C. Immunofluorescence staining for RUNX-2 (#sc10758; Santa Cruz Biotechnology, Heidelberg, Germany, dilution 1:100) was performed for 1 h. Isotype controls were performed with polyclonal rabbit isotype control antibody (Thermo Fisher Scientific, dilution 1:100). Subsequently, sections were incubated with anti-rabbit Alexa Fluor 594 secondary antibody (Thermo Fisher Scientific) for 1h at RT, performing DAPI counterstaining in parallel. For quantification, an inverse Olympus X83 microscope was used and the number of pixels in a region of interest were quantified using the software Photoshop (Adobe Systems, San Jose, California). Immunohistochemically positive stained pixels were automatically selected by using the Magic Wand Tool (settings: tolerance 20%; noncontiguous). Results are shown in % related to titanium.

### Alizarin Red (ARed) S staining

Material specimens were placed into wells of 12-well plates and seeded at a concentration of 1.25x10^5^ osteoblasts in 1 ml medium. Cells grown on the plastic cell culture plate served as positive controls. After 72 h, medium was changed by osteoblast mineralization medium for 1 week. ARed S Staining was performed according to the manufacturer’s instructions by ARed-Q (ScienCell Research Laboratories, CA, USA). For quantification, pictures were taken and the number of pixels in a region of interest was quantified using the software Photoshop (Adobe Systems, San Jose, California). Positive stained pixels were automatically selected by using the Magic Wand Tool (settings: tolerance 20%; noncontiguous). Results are shown in % related to titanium.

### Hemocompatibility

The study was reviewed and approved by the ethical committee of the Ruhr-University Bochum, Germany, with the registration number 16-5619-BR. All participants agreed by written informed consent. Investigation of blood compatibility of the specimens was performed according to the recommendations given by the standard protocol DIN ISO 10993–4 (Biological evaluation of medical devices–Part 4: Selection of tests for interactions with blood). All examinations from the blood collection to the final analysis were carried out within less than 2 hours. Whole blood samples were taken from three healthy volunteers in EDTA (for complete blood count (CBC)) or sodium citrate (all other tests) containing vials. A total of 2 specimens (dimensions 9.5 mm x 30 mm and 1 mm thickness) of the same alloy were placed in 40 cm polyvinyl chloride tubes (3/8”x 3/32“) with the „Ph. i. s. i. o.”(Phosphorylcholine inert surface) coating (Cormed Medizintechnik GmbH & Co. KG, Rüthen, Germany), a special polymer layer which hinds plasma protein deposition and platelet adhesion. Afterwards, 4 ml blood was added (= 3 cm^2^/ml surface/blood-ratio), the tube was circular bended and both ends were tightly closed by heat-shrink tubing. Tubes loaded with blood but without metal specimens served as negative controls. Tubes enriched with 0.4 ml blood and 3.6 ml ddH_2_O served as positive controls. Circular tubes were turned in a 38°C water bath for 60 min (resulting in a blood temperature of 37°C).

Metal specimens and blood were taken for further analyses of I) hemolysis, II) coagulation (partial thromboplastin time (PTT), fibrinogen-C concentration, International Normalized Ratio (INR) of prothrombin time), III) platelet activation (platelet count (% loss), platelet adhesion (SEM) and IV) Haematology (blood count). Analyses were performed utilitzing common clinical devices in the Institute of Clinical Chemistry, Transfusion and Laboratory Medicine, BG University Hospital Bergmannsheil, Ruhr-University Bochum, Bochum, Germany. SEM analyses of the metal specimens were performed as described in section 2.6., after carefully washing the specimens in PBS and fixation in 3% glutaraldehyde. Pictures of five randomly distributed regions of interest per specimen were taken (750x magnification) and platelets were counted.

### Nanoindentation and scratch test

To analyze the mechanical properties of the specimens, nanoindentation tests were performed on a nanoindentation module iMicro (Nanomechanics, Oak Ridge, TN, USA) equipped with a Berkovich diamond tip. Experiments were conducted with the continuous stiffness measurement method to determine indentation hardness H_IT_ as a function of indentation depth. A maximum load of 1000 mN with a loading and unloading rate of 0.2 s^-1^ was utilized. Hardness was calculated according to [23,24]. Since the hardness is not constant and dependent on indentation depth, hardness was averaged from 500 nm to 2500 nm for each measurement to obtain a single hardness value for each conducted indentation test.

Scratch tests were performed with a scratch tester (CSM instruments, Freiburg, Germany; NST module) equipped with a sphero-conical diamond (tip radius 2 μm) as previously described [25]. Test load parameters were gradually increasing (3 mN to 1000 mN) and scratch speed was set to 400 μm/min with a resulting scratch length of 400 μm. Measured scratch depth was plotted against scratch length. Resulting deformations were analyzed by SEM.

### Corrosion resistance test

Due to biomedical testing corrosion of material specimens was analyzed in MEM supplemented with 10% fetal bovine serum and penicillin/streptomycin, to have a surrounding microenvironment similar to human body physiology. Corrosion behavior of the four materials was investigated by polarization curves according to ASTM G5-94 [26], in order to measure the break-down-potential (E_bd_) and the repassivation/ protection-potential (E_prot_) of the materials. The tests were conducted with a three-electrode arrangement, consisting of an auxiliary (counter) electrode, a calomel reference electrode (Hg_2_Cl_2_, +243 mV to standard hydrogen electrode (SHE)) and a working electrode, which were connected to a potentiostat (PGZ 301, Radiometer Analytical, Lyon, France). The auxiliary electrode contains a platinized plate, which has an active surface of 1 cm². All electrochemical tests were carried out at 37°C inside a double walled glass vessel. At the beginning of the corrosion test, specimens were polarized with (1 mV/s) from -1970 mV to -2000 mV and back to -1970 mV followed by an open-circuit-potential-measurement (OCP) for 30 min. In the next step the potential was increased (forward curve) with 1 mV/s from -559 mV to 3741 mV for the TiAl6V4-sepcimens and from -559 mV to +2041 mV for all other specimens. After the maximum potential was reached, it was decreased (backward curve) with the same velocity until the initial potential of -559 mV. The intersection between forward and backward curve is defined as the protection potential.

### Statistical analysis

All experiments were repeated at least 3 times. Results of all experiments were given as mean ± SEM and compared with Titanium, the clinical gold standard, which was defined as 1 or 100% in all relative results. Statistical analyses were performed by unpaired 2-tailed student's t-test. P-values <0.05 were considered statistically significant and indicated in the figures as follows: *: p < 0.05; **: p < 0.01; ***: p < 0.001.

## Results

### Safe attachment and taintless vitality of cells

**Fig 1:**
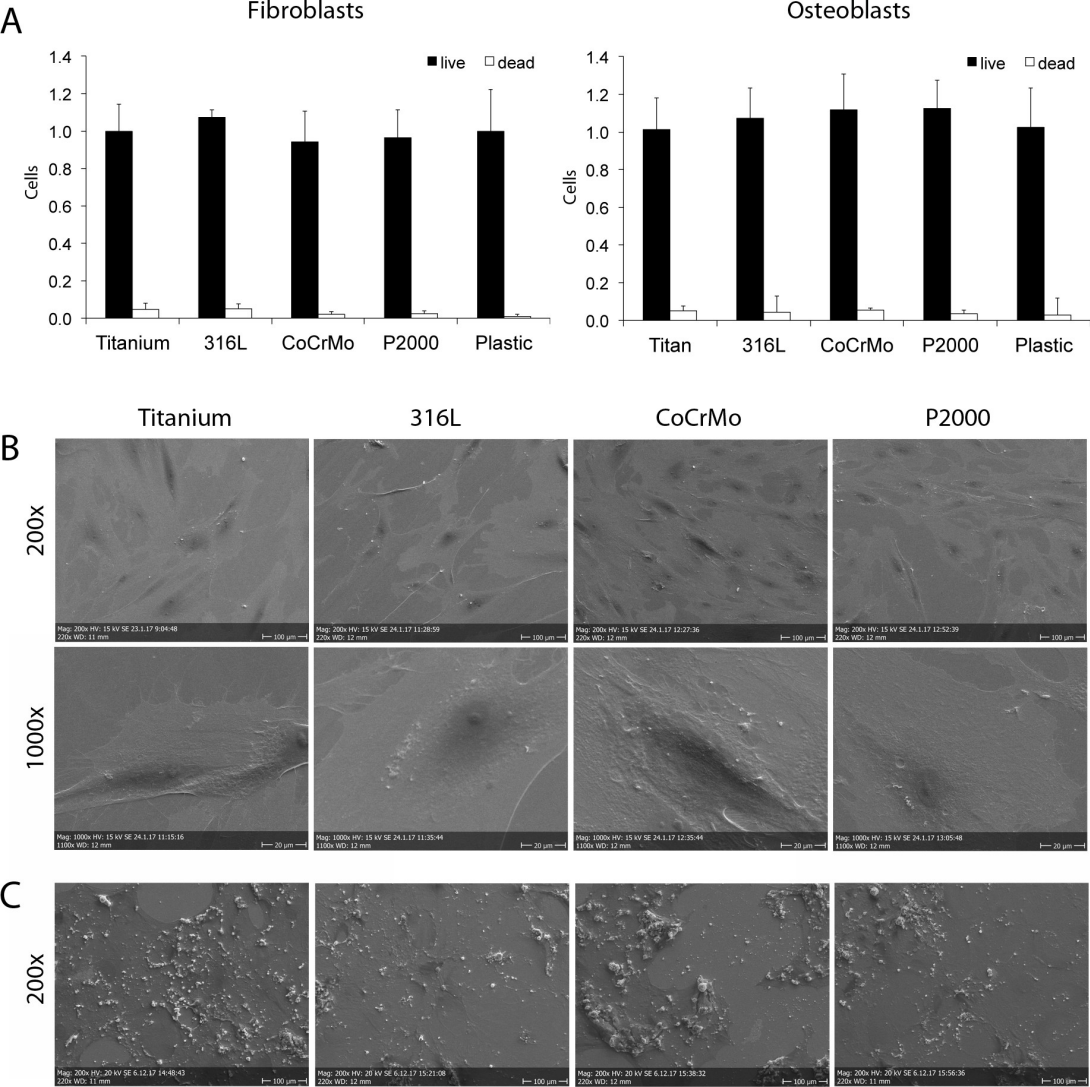
**Quantitative cell staining (A) and scanning electron microscopy (B,C).** Fibroblasts and osteoblasts grown for 24 h on different samples were live/dead stained with fluorescein diacetate (FDA) and propidium iodide (PI). Cells grown on plastic cell culture plate served as control. Data are presented as mean ± S.E.M (A). Scanning electron microscopic analyzes were performed with osteoblasts grown for 24 h on the samples and images were taken with a 200-fold and 1000-fold magnification. Representative images are shown (B). Additionally, images of osteoblasts cultivated in mineralization medium for 3 weeks were analyzed at a 200-fold magnification (C).

To investigate the vitality of fibroblasts and osteoblasts on P2000 compared to other alloys, live/dead stainings of the cells plated on titanium, 316L, CoCrMo, P2000 and control samples were performed. No significant differences in the amount of vital fibroblasts and osteoblasts were seen 24 h after cell seeding on all material specimens (Fig 1A). The amount of dead cells was negligibly small in all samples. Scanning electron microscopic analyzes revealed no marked abnormalities as to osteoblast attachment before (Fig 1B) and after mineralization (Fig 1C).

### Cytotoxicity and proliferation

**Fig 2:**
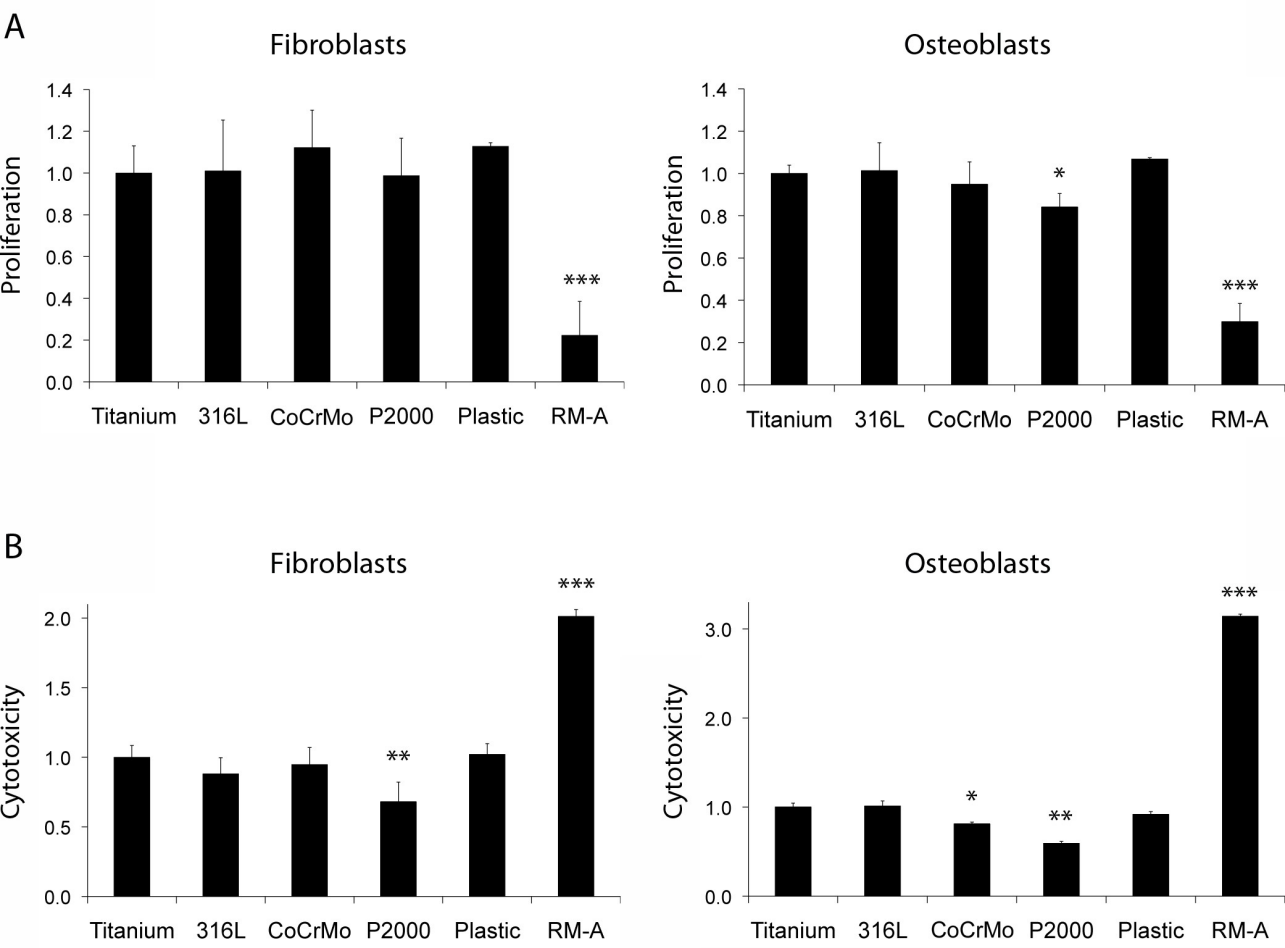
**XTT proliferation (A) and LDH cytotoxicity (B) assays.** Fibroblasts and osteoblasts grown for 24 h on different samples were stained. Cells were grown on plastic cell culture plates as positive controls and on RM-A sheets as negative controls. Data are presented as mean ± S.E.M. Student's t-test. (*p < 0.05, **p < 0.01, ***p < 0.001).

No significant differences in proliferation of fibroblasts were observed between samples. Osteoblast grown on P2000 showed a minimal reduction of cell proliferation (Fig 2A). A significant lower cytotoxicity for P2000 was observed in fibroblasts as well as in osteoblasts compared to titanium and all other samples (Fig 2B). Additionally, indirect XTT and LDH assays were performed with fibroblasts incubated with extracts of the material specimens for 24 h, according to the recommendations given by the standard protocol DIN ISO 10993–5. In comparison to the direct assays a significant lower cytotoxicity was observed for P2000 (S1 Fig).

### Osteoblastic activity

**Fig 3:**
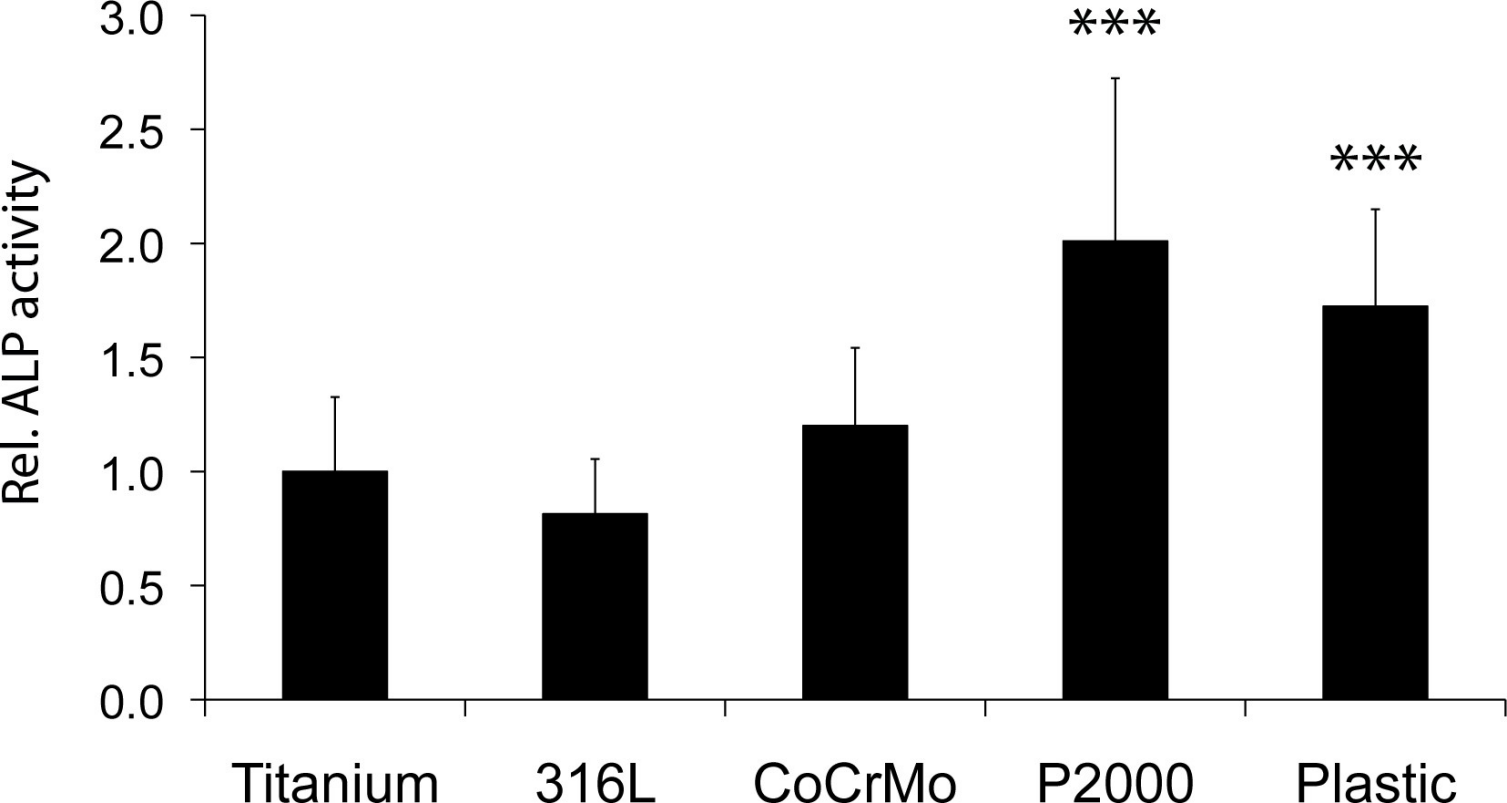
Alkaline phosphatase (ALP) activity. Osteoblasts grown for 1 week on different samples in mineralization medium were stained for ALP activity by a colorimetric test. Cells grown on plastic cell culture plate served as positive control. Cells on RM-A controls died completely after 1 week. Data are presented as mean ± S.E.M. Student's t-test. ***p < 0.001).

**Fig 4:**
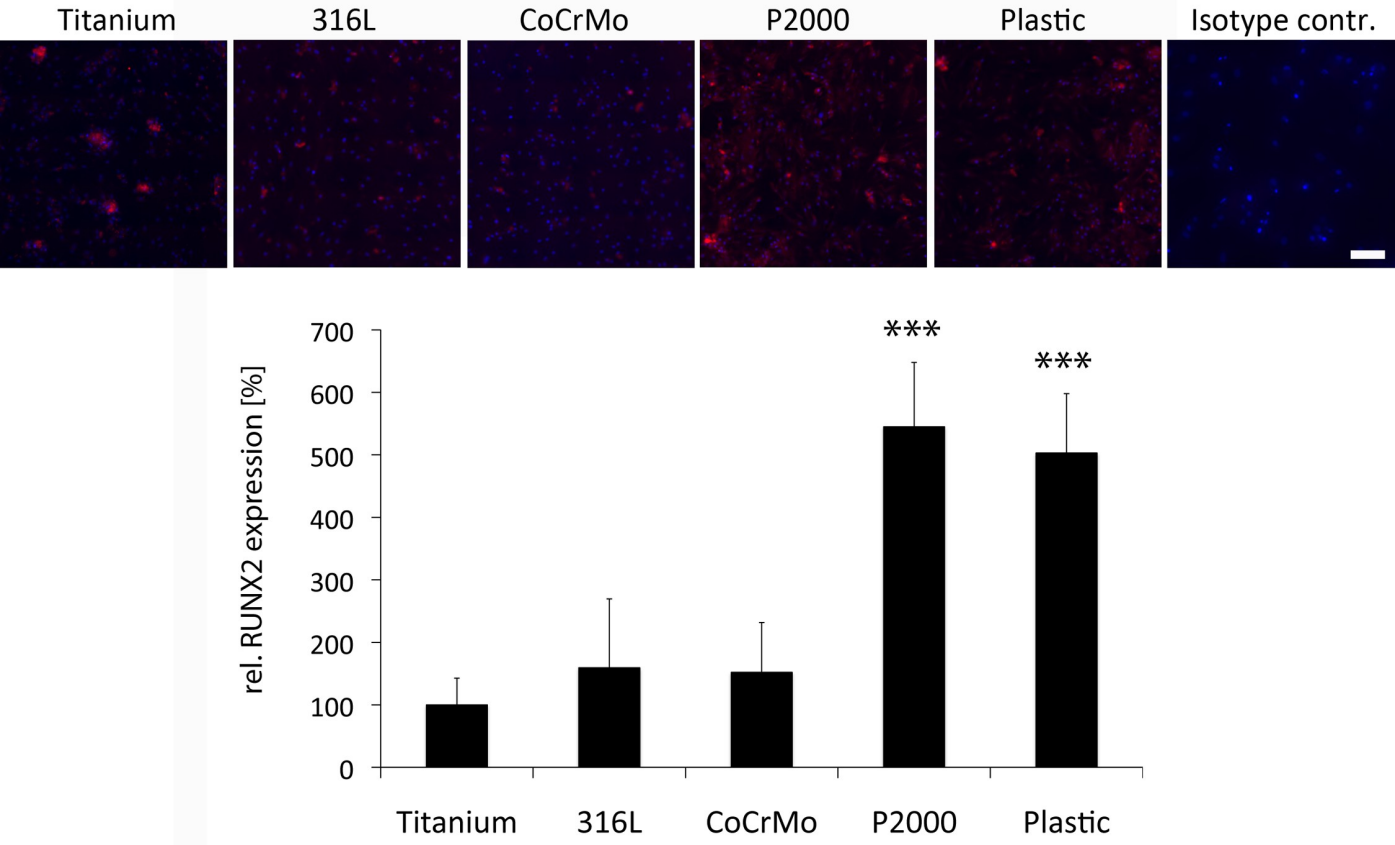
Immunofluorescence staining of RUNX2. Osteoblasts grown on different samples for 48 h in mineralization medium were stained. Cells on RM-A controls died after 48 h. Representative images are shown. Data are presented as mean ± S.E.M. Student's t-test. ***p < 0.001. Scale bar: 200 μm.

Alkaline phosphatase (ALP) activity, indicating osteoblast activity [27], showed a significant upregulation for P2000 and the plastic control (2.0 times and 1.7 times, respectively) as compared to titanium upon osteogenic differentiation of osteoblasts on the respective samples (Fig 3). Additionally, protein levels of Runt-related transcription factor 2 (*RUNX-2*), a key transcription factor regulating differentiation of osteoblasts [28,29], were investigated by immunofluorescence staining after 48 h. Compared to titanium, RUNX-2 protein levels for P2000 were approximately 5x higher (Fig 4).

### Biomineralization of osteoblasts

**Fig 5:**
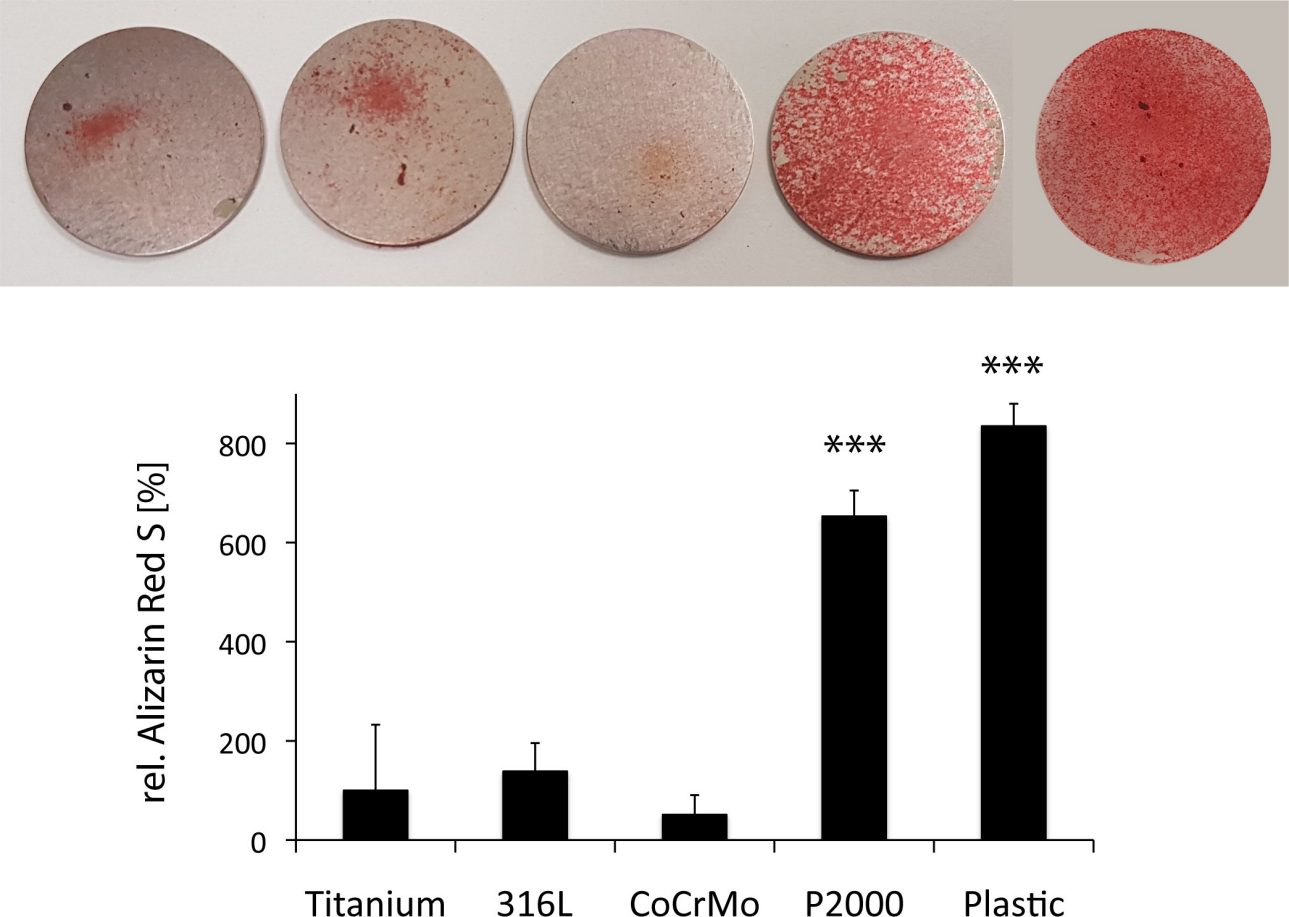
Alizarin Red S staining. Osteoblasts grown for 3 weeks on different samples in mineralization medium were stained with Alizarin Red S for biomineralization. Cells on RM-A controls died completely after 3 weeks. Data are presented as mean ± S.E.M. Student's t-test. ***p < 0.001.

In order to analyze osteoblast biomineralization on different materials, samples were stained with Alizarin Red S (Fig 5). For this purpose, osteoblasts were cultivated for 3 weeks on titanium, 316L, CoCrMo and P2000 specimens in mineralization medium. A significant increase of biomineralization was observed for osteoblasts grown on P2000 for 3 weeks (6.5x) as compared to titanium (Fig 5).

### Hemocompatibility

**Fig 6:**
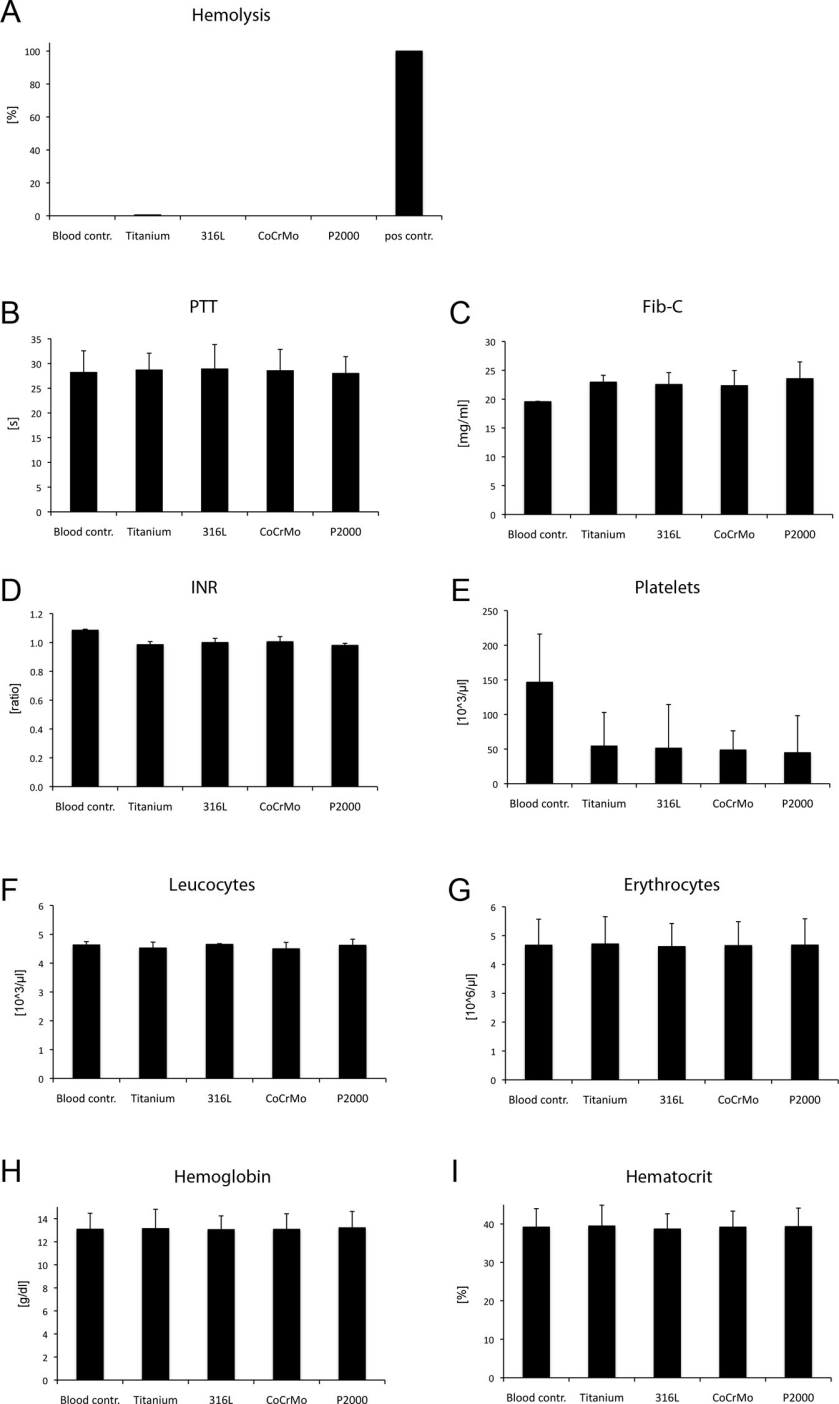
Blood compatibility assay. Testing of blood compatibility by the categories hemolysis (A), coagulation (PTT (B), Fib-C (C), INR (D)), platelet adhesion/activation (platelet count (E)), hematology (leucocytes (F), erythrocytes (G), hemoglobin (H), hematocrit (I)). All tests were performed 60 min after incubation in blood. Blood controls were tubes loaded with blood lacking a metal specimen (= negative control). Hemolysis is shown relatively to the positive control (tubes loaded with 0.4 ml blood and 3.6 ml ddH_2_O = 100%). Data are presented as mean ± S.E.M.

**Fig 7:**
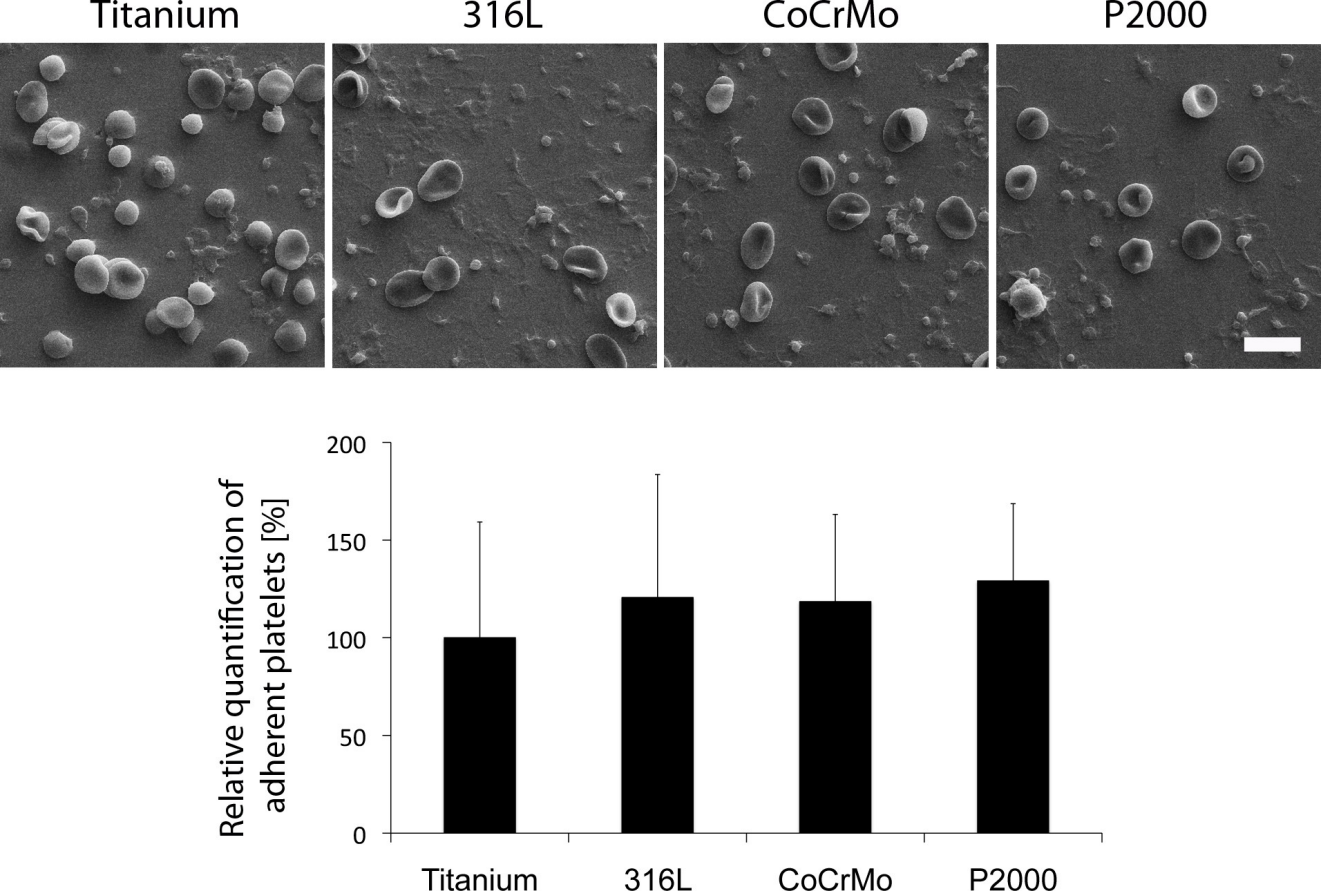
Testing of blood compatibility by platelet adhesion. After 60 min incubation in blood, metal specimens were fixed and analyzed by SEM. Representative images are shown. Data are presented as mean ± S.E.M. Scale bar demonstrates 10 μm.

No significant impact of P2000 on hemocompatibility was observed in any analyzed parameter compared with the other alloys (Fig 6). As expected, a decrease in platelet count was observed in all alloys compared with blood controls (tubes loaded with blood lacking a metal specimen) (Fig 6E). Additionally, no significant differences in the binding of platelets to the several alloys was observed by SEM analyses (Fig 7). A summary of the hemocompatibility testing results with determination of titanium as standard (= 100%) is shown in Table 1.

**Table 1:** Categories and results of blood interaction tests.

		Titanium	316L	CoCrMo	P2000	Contr.
Hemolysis	Material induced (%)	0.6	0	0	0	neg contr.: 0 pos. contr.: 100
Coagulation	PTT (%)	100	100.4 ±5.4	99.3 ±3.3	97.5 ±0.3	98.0 ±3.7
	Fib-C (%)	100	101.4 ±2.9	102.1 ±4.9	98.6 ±5.0	111.9 ±3.1
	INR (%)	100	101.5 ±0.7	102.0 ±1.4	99.5 ±0.7	110.2 ±1.7
Platelet activation	Count (%)	100	70.6 ±53.1	110.8 ±47.7	63.6 ±41.8	909.0 ±188.6
	Adhesion (%)	100	120.6 ±62.9	118.5 ±44.7	129.1 39.5	-
Haematology	LEU (%)	100	103.0 ±4.2	99.6 ±9.4	102.3 ±9.3	102.4 ±2.1
	ERY (%)	100	98.4 ±2.7	99.1 ±2.3	99.3 ±0.6	99.3 ±0.8
	HGB (%)	100	99.5 ±3.6	99.7 ±2.4	100.7 ±2.0	99.8 ±2.2
	HKT (%)	100	98.2 ±3.45	99.5 ±3.1	99.7 ±1.5	99.3 ±1.4

Controls were tubes loaded with blood lacking a metal specimen (= negative control), or tubes loaded with 0.4 ml blood and 3.6 ml ddH_2_O (= positive control, only for hemolysis experiments). Hemolysis is shown relatively to the pos. contr. (= 100%). All other tests are shown in relation to titanium (= 100%).

### Hardness and corrosion resistance

**Fig 8:**
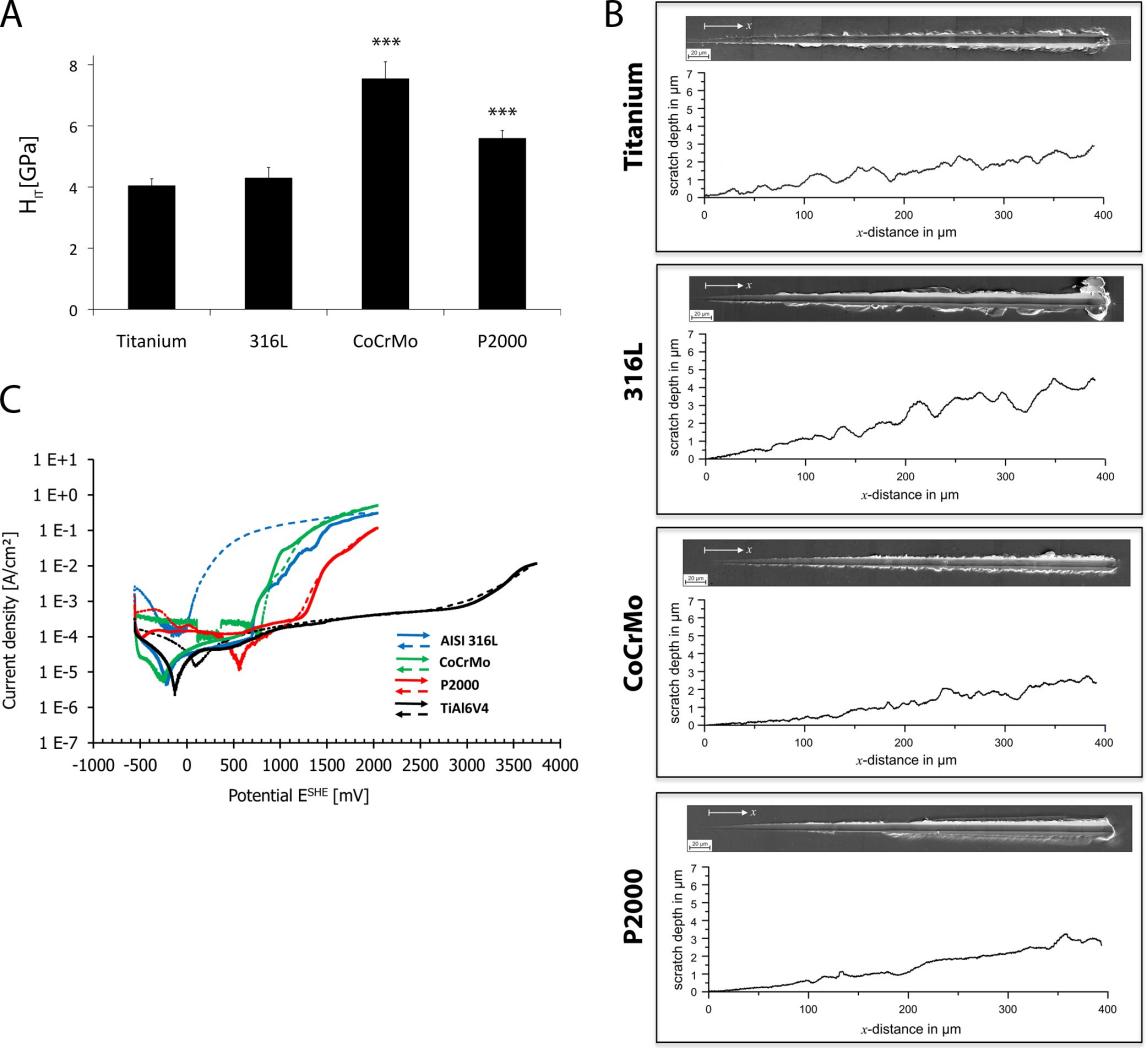
Electrochemical properties. Nanoindentation hardness test (A), scratch test (B) and corrosion resistance test (C) of four specimens. Data are presented as mean ± S.E.M. Student's t-test. (***p < 0.001).

To examine mechanical properties a nanoindentation hardness test was performed. Titanium and 316L specimens showed an indentation hardness (H_IT_) of about 4 GPa, whereas CoCrMo and P2000 revealed H_IT_ of 7.5 and 5.6 GPa, respectively (Fig 8A). Additional scratch tests exhibited differences of the deformation behavior. Titanium and 316L showed more irregular pile-ups at the edges of the groove compared to CoCrMo and P2000. Investigation of corrosion behavior revealed an improved corrosion resistance of nickel-free, high-nitrogen austenitic steel P2000 compared to 316L steel (Fig 8C and Table 2). Titanium showed the highest corrosion resistance.

**Table 2:** Summary of corrosion tests (Fig 8C).

	OCP* [mV]		i_P_* [A/cm²]		E_corr_ vs. SHE^**^ [mV]		E_bd_ vs. SHE^***^ [mV]		E_prot_ vs. SHE^****^ [mV]	
	mean	standard deviation	mean	standard deviation	mean	standard deviation	mean	standard deviation	mean	standard deviation
AISI 316L	-188	10	2.9E-05	4.2E-06	-223	14	no break-down		1110	78
CoCrMo	-323	14	3.5E-05	9.4E-06	-302	50	635	6	1009	178
P2000	-321	66	1.8E-04	7.0E-05	-365	174	1217	34	1110	78
TiAl6V4	-209	36	5.0E-05	1.3E-05	-82	35	3250	304	2910	396

*Open circuit potential

** Passive current density

*** Break-down potential

**** Protection potential

## Discussion

In this study, we could demonstrate that the nickel-free high-nitrogen steel P2000 is a suitable candidate for application in bone surgery. As to nearly all aspects like biocompatibility and hemocompatibility, cell metabolism, mineralization of osteoblasts and mechanical properties, P2000 was similar to or revealed advantages against titanium, 316L or CoCrMo. The capability of implant materials to promote new bone formation is of critical importance for supporting the healing of bone defects. Today, TiAl6V4 and 316L are the most common alloys for application in bone surgery, whereas both share advantages and disadvantages. The increased risk of cold welding and the high production costs of titanium on one hand, and the release of metal ions such as nickel by 316L steel on the other hand created needs to search for alternative alloys. Besides causing allergic reactions in patients, nickel has also been shown to inhibit the catalytic activity of enzymes (for example catalase [30] and glutathione reductase [31]). Referring to bone physiology, it has been demonstrated that catalytic activity of ALP is inhibited by metal ions like aluminum or nickel [32,33]. Moreover, nickel showed severe cytotoxicity toward osteoblast-like cells (SaOS2) [34]. Thus, nickel-free, high-Nitrogen austenitic steels like e.g. X13CrMnMo18-14-3 (brand name: P2000) appear as suitable candidates for an implant alloy in bone surgery.

In this study, we could demonstrate that P2000 revealed a safe attachment of fibroblasts and osteoblasts without compromises in vitality or changes of cell morphology. In a 1994 dated study it was claimed that osteoblasts cultured on titanium were significantly larger and were found to adhere in greater numbers to titanium compared with tissue culture polystyrene and CoCrMo [35]. This was not observed in our experiments. All alloys revealed similar vitality and morphology. Furthermore, a slight decrease in proliferation was observed for osteoblasts grown on P2000. This could be due to the initiation of the mineralization/differentiation process by P2000, which also inhibits the proliferation process [36]. However, the LDH assay exhibited lower cytotoxicity for P2000 compared to the other alloys and negative controls in direct and indirect contact tests (Figs 2 and S1). Probably, some components in P2000 could reduce LDH enzymatic activity and could distort results.

Moreover, depending on the implant material, osteoblastic activity was more or less impaired. Thus, investigation of the osteoblasts function by ALP activity and protein levels of RUNX2 revealed 2x increased ALP activity and more than 4x increased RUNX2 protein levels for P2000 compared to titanium or 316 steel, respectively (Figs 3 and 4). Runx2 is a key transcription factor regulating proliferation of osteoblast progenitors, differentiation of osteoblasts and mandatory for bone development [28, 29], hence chosen for analyzing osteoblastic activity in this study. The impact of the implant material on the osteoconductivity was also demonstrated in previous studies. Copper-bearing medical 317L steel (317L-Cu) that released a trace amount of Cu^2+^ ions to the physiological environment promoted adhesion and proliferation and RUNX2 gene expression of osteoblasts cultured on its surface more than untreated 317L steel [37]. Increased ALP activity was also observed after incubation of osteoblasts on 317L-Cu [38]. Additionally, in vivo tests with rats demonstrated more new bone tissue formation around the 317L-Cu implant with stable bone-to-implant contact and a higher bone mineral density (BMD) [37]. Likewise, strontium (Sr) or zinc (Zn) releasing titanium implants also accelerated new bone formation in rats or rabbits and increased bone ingrowths of implants [39,40], whereas reduction of osteogenic response of osteoblast-like SaOS-2 cells was observed after exposure to cobalt (Co^2+^) and chromium ions (Cr^3+^) [41]. Moreover, surface modifications with TiO_2_ nanotubes also showed elevated ALP activities [42,43]. These references confirm the important influence of implant materials on osteoconductivity. In our study P2000 showed the highest bioactivity towards osteoblasts. Which specific chemical components of P2000 are responsible for this process must be investigated in further studies.

Supporting the results of increased ALP activity and RUNX2 levels, enhanced biomineralization of osteoblasts grown on P2000 were observed by Alizarin Red S. Comparable to results shown in this manuscript, a previous study demonstrated the influence of surface chemistry on osteoblastic differentiation and matrix mineralization independent from alterations in cell proliferation [44]. Four different gold alkanethiol surfaces with OH-, NH2-, COOH- and CH3- termination were synthesized and compared for osteoconductivity. OH- and NH2-terminated surfaces revealed an up-regulation of osteoblast-specific gene expression, ALP activity, and matrix mineralization compared with surfaces presenting COOH- and CH3- groups [44]. Thus release of nitrogen could be a possible reason for the good osteoconductivity of P2000.

The DIN ISO 10993–4 blood compatibility tests demonstrated that P2000 has no hemolytic effect and no significant influence on hemocompatibility and, therefore, it proves being a safe alloy for application in bone surgery. Platelet counts were decreased in all tested alloys compared to blood controls. However, this does not account for negative blood compatibility of P2000, since titanium and steel showed similar platelet counts (Fig 6E). Further adhesion tests exhibited also comparable amounts of adherent platelets and no significant morphological differences of platelets on distinct alloys. Importantly, the affinity of platelets to metal surfaces and the formation of pseudopodia-like structures is a known phenomenon [45,46].

The wear resistance of solution annealed P2000 under boundary lubricated sliding wear in proteinaceous media being comparable to that of CoCrMo and better than 316L has been published earlier [47]. This has been related to the distinct toughness in combination with the cold working capability under plastic shear. Scratch tests support this by showing homogenous generation of pile-ups at the edges of the scratch in contrast to those of the other alloys tested. Irregular pile-ups are characteristic for a less ductile behavior and are prone to transform into chip-like wear particles. Moreover, an improved corrosion resistance of P2000 compared to 316L steel was investigated, whereas titanium showed the highest corrosion resistance. It is known, that nitrogen (N) additions improve the corrosion resistance of austenitic stainless steels [48].

A limitation of the study is that we focused on the biomaterial´s influence on osteoblasts and its biocompatibility. Further experiments have to analyze the interaction of P2000, osteoclastogenesis and inflammatory reaction as biomaterial-derived debris is incorporated by macrophages. Polarization of macrophages towards an inflammatory M1 type or anti-inflammatory M2 type is dependent on MSCs [49]. Finally, a stimulation of osteoclast can cause particle-induced osteolysis and loosening of the implant in the long term.

## Conclusions

In summary, this study demonstrates the advantages of the nickel-free, high-nitrogen austenitic steel P2000, as to good biocompatibility and hemocompatibility, increased osteogenesis and mineralization compared to TiAl6V4, 316L or CoCrMo. Furthermore, P2000 performed improved mechanical properties as to strength and toughness, and high corrosion resistance. P2000 exhibited a safe attachment of cells without compromises in vitality or changes of cell morphology. Additionally, increased osteoblasts function was observed for P2000 compared to titanium or 316 steel, investigated by ALP activity and protein levels of RUNX2. Moreover, enhanced biomineralization of osteoblasts grown on P2000 were observed. Furthermore, an improved corrosion resistance of P2000 compared to 316L steel was investigated in microenvironment similar to human body physiology. P2000 is available and has a processing similar to that of other steels, thus can therefore be used directly. Thus P2000 appears to be a promising alternative candidate for applications in bone surgery. The present study provides significant data to support *in vivo* trials, thus further *in vivo* studies are in progress.

## Supporting information

S1 Fig **Indirect XTT proliferation (A) and LDH cytotoxicity (B) assays. Fibroblasts incubated with extracts of the material specimens for 24 h were stained.** Cells were incubated with extracts of plastic cell culture plates and RM-A sheets as controls. Data are presented as mean ± S.E.M. Student's t-test. (*p < 0.05, **p < 0.01, ***p < 0.001).
(TIF)

S1 Dataset Supporting information containing the dataset used for the figures in the manuscript. (PDF)
