# Supplementary figures and images for: P2000 - A high-nitrogen austenitic steel for application in bone surgery
Source: PLoS One. 2019 Mar 26;14(3):e0214384. doi: 10.1371/journal.pone.0214384 (PMC6435142; doi:10.1371/journal.pone.0214384)

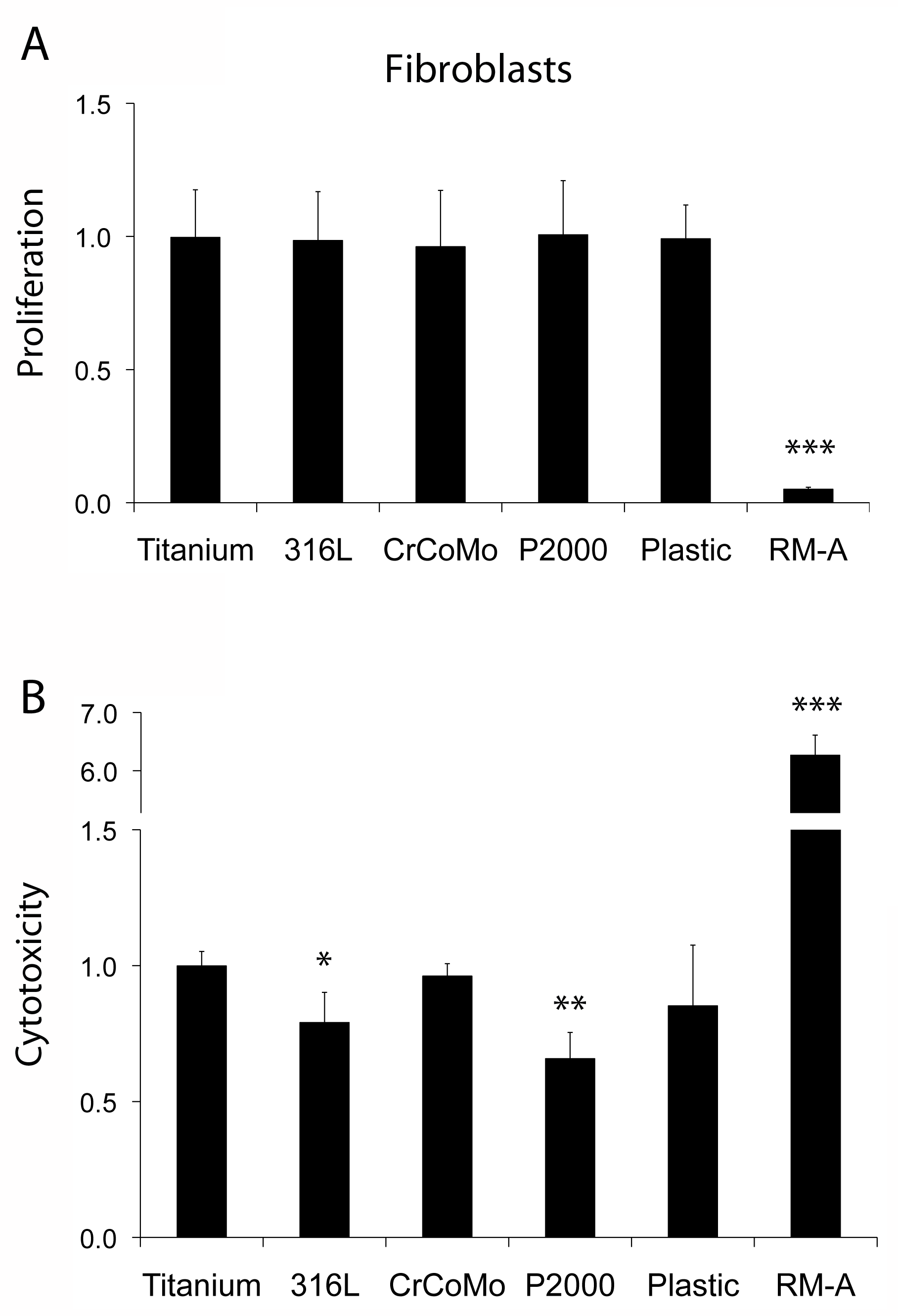

Supplement: S1 Fig — Indirect XTT proliferation (A) and LDH cytotoxicity (B) assays. Fibroblasts incubated with extracts of the material specimens for 24 h were stained. Cells were incubated with extracts of plastic cell culture plates and RM-A sheets as controls. Data are presented as mean ± S.E.M. Student's t-test. (*p < 0.05, **p < 0.01, ***p < 0.001). (TIF) [file pone.0214384.s001.tif]
