# Supplementary material for: P2000 - A high-nitrogen austenitic steel for application in bone surgery
Source: PLoS One. 2019 Mar 26;14(3):e0214384. doi: 10.1371/journal.pone.0214384 (PMC6435142; doi:10.1371/journal.pone.0214384)

XTT L929 direct

	#1				#2				#3				#4				#5			
**Titan**	0,699	0,693	0,594	0,654	0,726	0,628	0,665	0,643	0,608	0,533	0,694	0,668	0,642	0,961	0,636	0,647	0,718	0,758	0,786	0,78
**316L**	0,493	0,535	0,608	0,612	0,663	0,524	0,584	0,467	1,129	0,957	0,947	0,743	0,801	0,705	0,671	0,594	0,687	0,771	0,749	0,629
**CoCrMo**	0,774	0,61	0,61	0,566	0,708	0,71	0,666	0,579	0,956	0,912	1,027	0,787	0,795	0,872	0,812	0,79	0,768	0,823	0,807	0,83
**P2000**	0,571	0,53	0,494	0,535	0,654	0,666	0,595	0,573	0,939	0,897	0,877	0,806	0,685	0,665	0,618	0,62	0,728	0,704	0,7	0,701
**RM-A**	0,149	0,152	0,151	0,15	0,145	0,14	0,147	0,126	0,176	0,173	0,162	0,166	0,151	0,152	0,151	0,152				
**Plastic**	0,725	0,773	0,702	0,729	0,722	0,675	0,696	0,602	0,925	0,941	0,849	0,913	0,595	0,908	0,814	0,823				

	**MW**	**Std**
**Titan**	0,687	0,090
**316L**	0,693	0,168
**CrCoMo**	0,770	0,123
**P2000**	0,678	0,123
**Plastic**	0,771	0,110
**RM-A**	0,153	0,014

	**MW**	**Std**
**Titan**	1,00	0,13
**316L**	1,01	0,24
**CrCoMo**	1,12	0,18
**P2000**	0,99	0,18
**Plastic**	1,12	0,16
**RM-A**	0,22	0,02

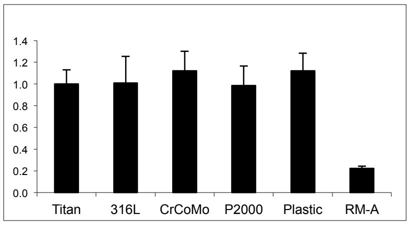

LDH L929 direct

Plate Title	#1				#2				#3			
	1	2	3	4	5	6	7	8	9	10	11	12
**Titan**	0,206	0,217	0,222	0,222	0,213	0,213	0,217	0,217	0,17	0,184	0,186	0,191
**316L**	0,195	0,201	0,21	0,183	0,194	0,19	0,194	0,198	0,138	0,146	0,153	0,164
**CrCoMo**	0,2	0,207	0,208	0,214	0,208	0,201	0,209	0,233	0,154	0,155	0,166	0,175
**P2000**	0,168	0,177	0,176	0,187	0,126	0,133	0,125	0,129	0,107	0,12	0,114	0,112
**RM-A**	0,383	0,426	0,413	0,427	0,408	0,408	0,402	0,432	0,411	0,43	0,396	0,415
**Plastic**	0,2	0,207	0,203	0,229	0,199	0,207	0,196	0,211	0,202	0,214	0,221	0,221

	MW	Std
**Titan**	0,205	0,017
**316L**	0,181	0,024
**CrCoMo**	0,194	0,025
**P2000**	0,140	0,029
**Plastic**	0,209	0,010
**RM-A**	0,413	0,015

	MW	Std
**Titan**	1,00	0,09
**316L**	0,88	0,12
**CrCoMo**	0,95	0,12
**P2000**	0,68	0,14
**Plastic**	1,02	0,05
**RM-A**	2,01	0,07

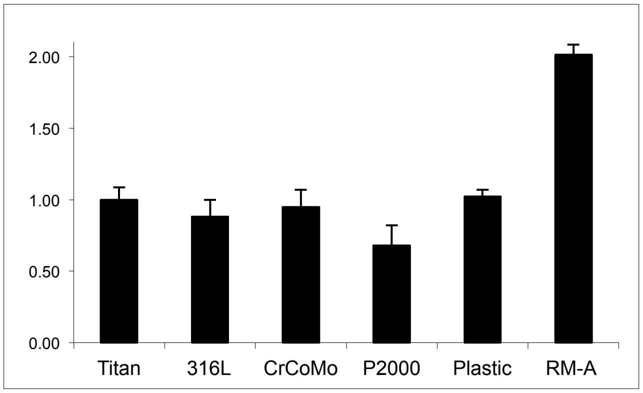

XTT Osteo direct

Plate Title		Titan	316L	CrCoMo	P2000	RM-A	Plastic	
	1	2	3	4	5	6	7	8
A	#1	0,949	0,955	0,775	0,882	0,205	1,045	
B		0,990	1,221	1,053	0,922	0,221	1,242	
C		1,040	1,031	0,990	0,850	0,216	1,283	
D		1,048	0,955	0,953	0,916	0,212	1,200	
E	#2	0,996	1,321	1,080	0,785	0,208	1,183	
F		1,025	1,208	0,915	0,783	0,220	1,087	
G		1,073	1,114	0,825	0,767	0,216	1,137	
H		0,991	1,035	0,866	0,778	0,204	1,056	
	#3	1,011	0,815	1,069	0,979	0,474	0,883	
		0,939	0,861	0,987	0,843	0,464	0,895	
		0,955	0,806	0,913	0,783	0,465	0,851	
		0,930	0,782	0,910	0,763	0,467	0,900	

	MW	Std			MW	Std
**Titan**	1,00	0,05		Titanium	1,00	0,05
**316L**	1,01	0,18		316L	1,01	0,18
CoCrMo	0,94	0,10		CoCrMo	0,95	0,10
**P2000**	0,84	0,07		P2000	0,84	0,07
**RM-A pos**	0,30	0,13		Plastic	1,07	0,15
**Plastic**	1,06	0,15		RM-A	0,30	0,13

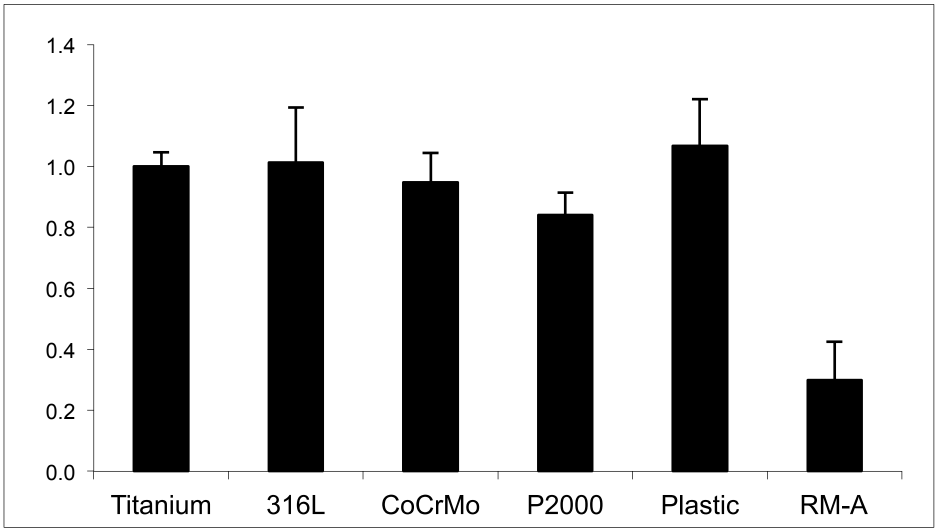

LDH Osteo direct

Plate Title		Titan	316L	CrCoMo	P2000	RM-A	Plastic	
	1	2	3	4	5	6	7	8
A	#1	0,202	0,21	0,164	0,126	0,59	0,175	
B		0,205	0,215	0,165	0,132	0,593	0,181	
C		0,209	0,207	0,16	0,128	0,594	0,181	
D		0,209	0,213	0,166	0,127	0,596	0,179	
E	#2	0,179	0,185	0,169	0,113	0,61	0,183	
F		0,192	0,186	0,163	0,118	0,612	0,188	
G		0,186	0,182	0,157	0,111	0,624	0,184	
H		0,192	0,194	0,159	0,115	0,617	0,19	
	#3	0,204	0,201	0,169	0,123	0,608	0,174	
		0,211	0,195	0,170	0,128	0,595	0,188	
		0,208	0,200	0,160	0,119	0,605	0,194	
		0,200	0,198	0,167	0,125	0,597	0,180	

	MW	Std
**Titan**	0,200	0,010
**316L**	0,199	0,011
**CrCoMo**	0,164	0,004
**P2000**	0,122	0,007
**RM-A**	0,603	0,011
**Plastic**	0,183	0,006

	MW	Std
Titanium	1,00	0,05
316L	1,00	0,06
CrCoMo	0,82	0,02
P2000	0,61	0,03
Plastic	0,92	0,03
RM-A	3,03	0,05

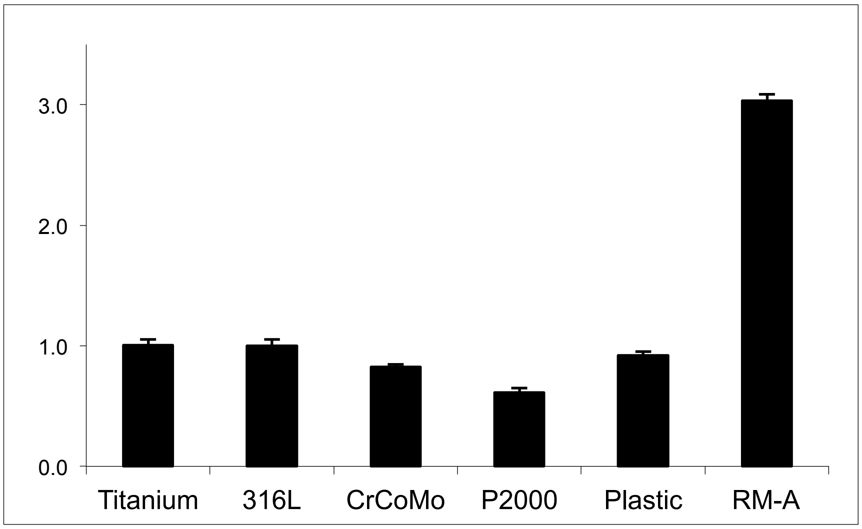

XTT L929 indirect

Plate Title		Titan	316L	CrCoMo	P2000	RM-A	Plastic	
	1	2	3	4	5	6	7	8
A	#1	2,849	2,667	1,817	2,518	0,116	2,666	
B		2,569	2,637	2,748	2,613	0,127	2,493	
C		2,649	1,995	2,011	2,07	0,128	2,54	
D		2,182	2,025	2,046	2,086	0,164	1,945	
E	#2	2,254	2,177	2,023	1,962	0,161	2,666	
F		2,211	2,503	2,534	2,785	0,133	3,016	
G		3,683	3,608	3,492	3,813	0,165	3,082	
H		3,556	2,672	2,608	2,667	0,123		
	#3	2,477	2,777	2,621	3,058	0,155	2,576	
		2,491	2,901	3,218	3,276	0,129	3,199	
		3,000	3,081	3,333	3,183		2,862	
		2,912	3,412	3,230	3,130		2,904	

	MW	Std
**Titan**	2,736	0,492
**316L**	2,705	0,504
**CrCoMo**	2,640	0,581
**P2000**	2,763	0,559
**RM-A**	0,140	0,019
**Plastic**	2,723	0,348

	MW	Std
**Titan**	1,00	0,18
**316L**	0,99	0,18
**CrCoMo**	0,96	0,21
**P2000**	1,01	0,20
**Plastic**	1,00	0,13
**RM-A**	0,05	0,01

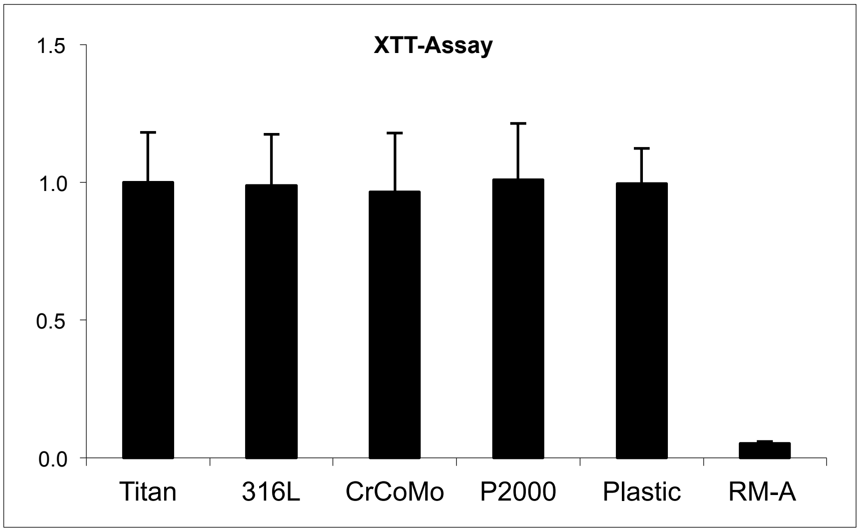

LDH L929 indirect

Plate Title		Titan	316L	CrCoMo	P2000	RM-A	Plastic	
	1	2	3	4	5	6	7	8
A	#1	0,217	0,182	0,218	0,15	1,378	0,205	
B		0,204	0,175	0,206	0,144	1,455	0,21	
C		0,214	0,171	0,209	0,151	1,339	0,207	
D		0,232	0,157	0,208	0,159	1,342	0,197	
E	#2	0,216	0,191	0,194	0,15	1,361	0,167	
F		0,211	0,197	0,215	0,148	1,352	0,17	
G		0,225	0,193	0,231	0,079	1,345	0,185	
H		0,248	0,238	0,206	0,137	1,556	0,035	
	#3	0,230	0,157	0,231	0,168	1,299	0,219	
		0,230	0,154	0,214	0,155	1,332	0,215	
		0,227	0,157	0,220	0,156	1,327	0,222	
		0,220	0,155	0,209	0,152	1,323	0,214	
		0,209	0,155	0,214	0,149	1,31	0,213	

	MW	Std
**Titan**	0,222	0,012
**316L**	0,176	0,025
**CrCoMo**	0,213	0,010
**P2000**	0,146	0,021
**RM-A**	1,363	0,070
**Plastic**	0,189	0,050

	MW	Std
**Titan**	1,00	0,05
**316L**	0,79	0,11
**CrCoMo**	0,96	0,05
**P2000**	0,66	0,10
**Plastic**	0,85	0,22
**RM-A**	6,14	0,31

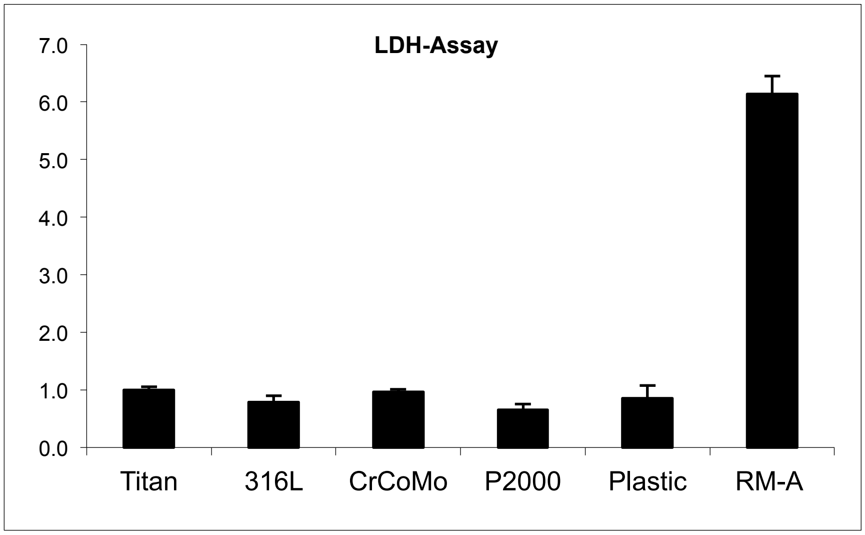

LiveDead L929

Live

**Titan**	Wert	MW	**316L**	Wert	MWMW	**CoCrMo**	Wert	MWMW	**P2000**	Wert	MWMW	**Plastic**	Wert	MWMW
#1	53,4	45,3	#1	46,5	48,3	#1	52,9	42,6	#1	48,5	43,8	#1	45,1	45,7
#2	50,5	Stabd	#2	47,8	Stabd	#2	37,2	Stabd	#2	35,2	Stabd	#2	35,9	Stabd
#3	43,9	6,6	#3	51,2	1,9	#3	38,1	7,5	#3	46,7	6,7	#3	56,1	10,1
#4	41,4		#4	47,0		#4	36,6		#4	38,2				
#5	37,3		#5	48,9		#5	48,5		#5	50,3				

Dead

**Titan**	Wert	MW	**316L**	Wert	MW	**CoCrMo**	Wert	MW	**P2000**	Wert	MW	**Plastic**	Wert	MW
#1	3,4	2,5	#1	4,5	2,8	#1	1,1	1,1	#1	0,8	1,0	#1	0,8	0,5
#2	1,1	Stabd	#2	1,7	Stabd	#2	1,4	Stabd	#2	0,5	Stabd	#2	0,4	Stabd
#3	2,1	1,3	#3	1,4	1,3	#3	1,3	0,4	#3	1,8	0,6	#3	0,2	0,3
#4	4,3		#4	3,5		#4	0,5		#4	1,2				
#5	1,5		#5	3,0		#5	1,4		#5	0,5				

Live	MW	Stabw
**Titan**	45,3	6,6
**316L**	48,3	1,9
**CoCrM**	42,6	7,5
**P2000**	43,8	6,7
**neg**	45,7	10,1
**Titaniu**	1,00	0,15
**316L**	1,07	0,04
**CoCrM**	0,94	0,17
**P2000**	0,97	0,15
**Plasti**	1,01	0,22

Dead	MW	Stabw
**Titan**	2,5	1,3
**316L**	2,8	1,3
**CoCrMo**	1,1	0,4
**P2000**	1,0	0,6
**neg**	0,5	0,3
**Titaniur**	0,05	0,03
**316L**	0,06	0,03
**CoCrMo**	0,03	0,01
**P2000**	0,02	0,01
**Plastic**	0,01	0,01

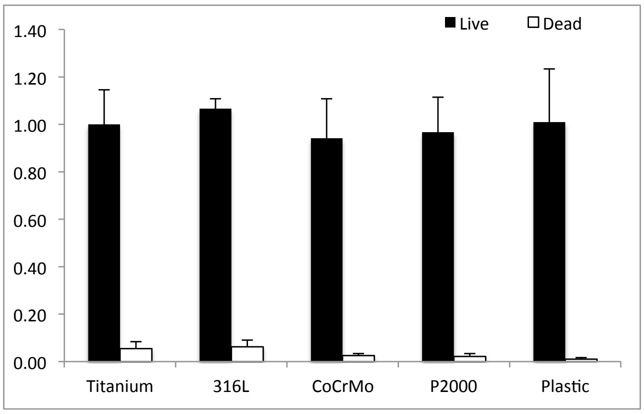

LiveDead Osteo

Live

**Titan**	Wert	MW	**316L**	Wert	MWMW	**CoCrMo**	Wert	MWMW	**P2000**	Wert	MWMW	Plastic	Wert	MWMW
#1	38,7	48,8	#1	46,2	52,0	#1	42,7	54,4	#1	46,1	54,4	#1	59,4	50,3
#2	59,1	Stabd	#2	57,9	Stabd	#2	65,1	Stabd	#2	53,0	Stabd	#2	41,6	Stabd
#3	46,4	8,5	#3	51,9	5,9	#3	56,7	9,3	#3	55,1	7,2	#3	49,9	8,9
#4	51,1					#4	53,2		#4	63,6				

Dead

**Titan**	Wert	MW	**316L**	Wert	MW	**CoCrMo**	Wert	MW	**P2000**	Wert	MW	Plastic	Wert	MW
#1	1,3	2,5	#1	8,5	2,6	#1	2,1	2,1	#1	2,4	2,0	#1	6,4	2,2
#2	3,7	Stabd	#2	0,1	Stabd	#2	2,4	Stabd	#2	1,5	Stabd	#2	0,0	Stabd
#3	2,5	1,3	#3	0,4	3,6	#3	2,3	0,4	#3	2,8	0,8	#3	0,1	3,7
#4	3,8		#4	3,5		#4	1,5		#4	2,2				
#5	1,2		#5	0,3		#5	2,4		#5	0,9				

Live	MW	Stabw
**Titan**	48,8	8,5
**316L**	52,0	5,9
**CoCrN**	54,4	9,3
**P2000**	54,4	7,2
**neg**	50,3	8,9
**Titaniu**	1,00	0,18
**316L**	1,07	0,12
**CoCrN**	1,12	0,19
**P2000**	1,12	0,15
**Plastic**	1,03	0,18

Dead	MW	Stabw
**Titan**	2,5	1,3
**316L**	2,6	3,6
**CoCrMo**	2,1	0,4
**P2000**	2,0	0,8
**neg**	2,2	3,7
**Titaniur**	0,05	0,03
**316L**	0,05	0,07
**CoCrMo**	0,04	0,01
**P2000**	0,04	0,02
**Plastic**	0,04	0,07

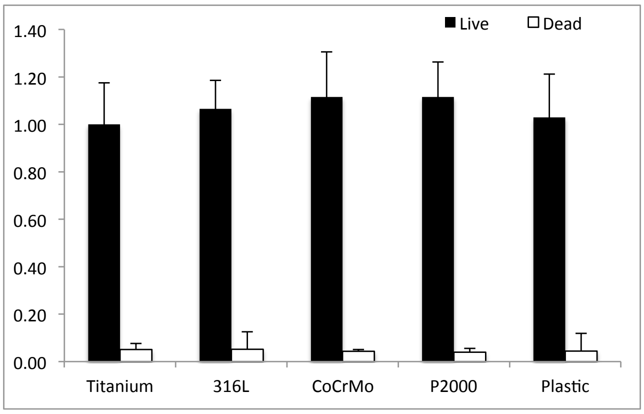

ALP Assay

	#1				#2				#3				#4			
**Titanium**	0,985	1,014	0,999	0,999	0,985	0,992	1,029	0,999	0,455	0,470	0,485		1,424	1,311	1,447	1,409
**316L**	0,634	0,634	0,650	0,642	0,867	0,859	0,850	0,925	1,246	1,087	1,110	1,208	0,598	0,542	0,572	0,598
**CoCrMo**	0,910	0,918	0,948	0,956	1,017	1,039	1,047	1,039	1,774	1,734	1,785	1,769	1,136	1,014	1,065	1,056
**P2000**	1,655	1,715	1,764	1,744	1,616	1,606	1,665	1,596	1,456	1,521	1,546	1,580	3,146	2,920	2,991	3,641
**Plastic**	1,470	1,544	1,497	1,497	1,222	1,229	1,242	1,202	1,930	2,075	2,243	2,037	2,192	2,100	2,386	

	MW	Stabw
**Titanium**	1,000	0,33
**316L**	0,814	0,24
**CoCrMo**	1,201	0,34
**P2000**	2,010	0,71
**Plastic**	1,724	0,43

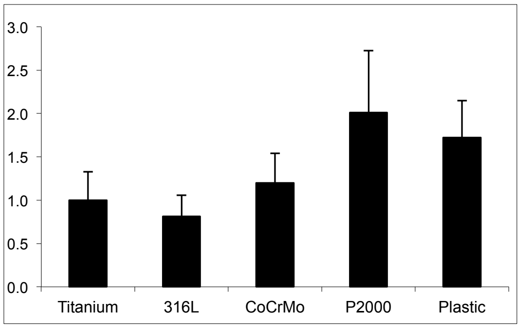

Alizarin

	#1	#2	#3	MW	STABW
Titanium	7218	305	38250	15258	20210
316L	11257	27825	24298	21127	8727
CoCrMo	10750	876	11761	7796	6014
P2000	90993	106567	101353	99638	7927
Plastic	121748	125776	135340	127621	6981

	MW	STABW
Titanium	100	132
316L	138	57
CoCrMo	51	39
P2000	653	52
Plastic	836	46

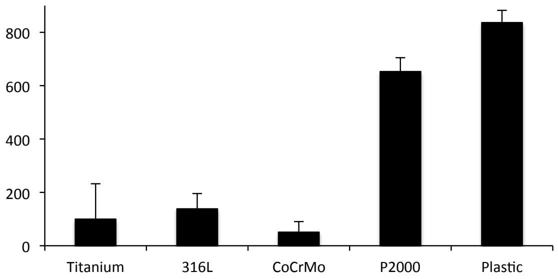

RUNX2

**Titanium**		**MW**	**Stabw**
**#1**	3935	6644	2875
	6691		
	3233		
**#2**	6742		
	10340		
	2846		
**#3**	10731		
	7226		
	8055		

**316L**		**MW**	**Stabw**
**#1**	15428	10706	7480
	21886		
	11622		
**#2**	2018		
	2530		
	3348		
**#3**	13266		
	6618		
	19638		

**Plastic**		**MW**	**Stabw**
**#1**	22759	33532	6389
	28090		
	35577		
**#2**	39634		
	41509		
	37231		
**#3**	38002		
	31675		
	27309		

**CoCrMo**		**MW**	**Stabw**
**#1**	16481	10075	5306
	12139		
	9860		
**#2**	10998		
	16208		
	13945		
**#3**	2567		
	3244		
	5234		

**P2000**		**MW**	**Stabw**
**#1**	27952	36206	6982
	32701		
	43946		
**#2**	44301		
	32265		
	29840		
**#3**	42701		
	29525		
	42626		

	MW	Stabw
**Titaniu**	6644	2875
**316L**	10706	7480
**CoCrM**	10075	5306
**P2000**	36206	6982
**Plastic**	33532	6389

	MW	Stabw
Titaniur	100	43
316L	161	113
CoCrMo	152	80
P2000	545	105
Plastic	505	96

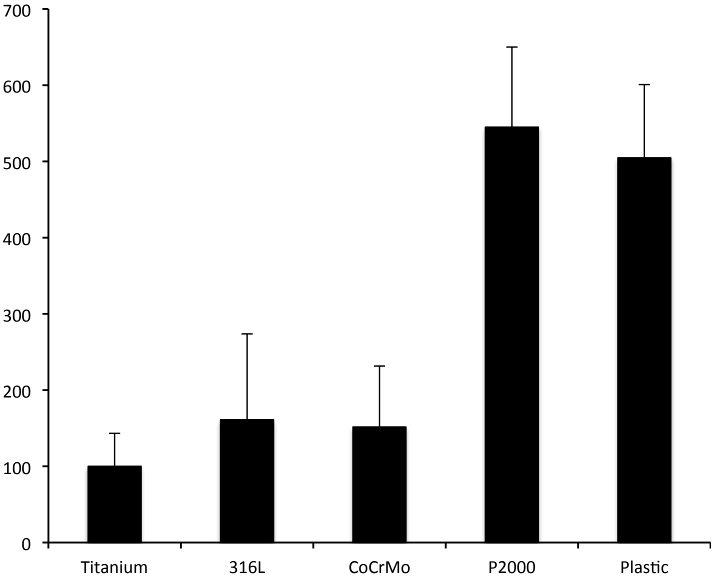

Blood

		blood contr	Ti	316L	CoCrMo	P2000	blood contr	Ti	316L	CoCrMo	P2000	Lysis contr
**Quick**	s	12,30	11,40	11,60	11,70	11,30	12,10	10,90	11,00	11,00	10,90	
	%	88,00	100,00	97,00	95,00	101,00	88,00	105,00	103,00	103,00	105,00	
	INR	1,09	1,00	1,02	1,03	0,99	1,08	0,97	0,98	0,98	0,97	
**PTT**	s	25,10	26,30	25,40	25,50	25,60	31,30	31,10	32,40	31,60	30,40	
**Fib-C**	s	16,20	14,20	14,10	14,00	13,50	15,90	14,50	15,00	15,30	14,80	
	mg/dl	196,00	238,00	240,00	242,00	256,00	195,00	221,00	211,00	205,00	215,00	
**Ddim**	µg/ml	0,15	0,14	0,25	0,27	0,24	0,21	0,25	0,21	0,23	0,24	
	mAbs	0,96	0,86	3,41	3,90	3,16	2,49	3,82	2,41	3,17	3,27	
**PTZ5**	s	16,30	12,40	12,80	12,90	12,50	15,20	12,30	12,20	12,30	11,90	
**LEU**	10^3/µl	4,71	4,67	4,67	4,34	4,47	4,55	4,38	4,64	4,65	4,77	
**ERY**	10^6/µl	4,04	4,04	4,06	4,07	4,03	5,31	5,38	5,19	5,24	5,32	
**HGB**	g/dl	12,11	11,95	12,20	12,12	12,20	14,06	14,32	13,89	14,03	14,21	
**HKT**	%	35,76	35,65	35,89	36,24	35,91	42,58	43,30	41,48	42,12	42,70	
**MCV**	fL	88,60	88,19	88,51	89,03	89,05	80,23	80,49	79,96	80,35	80,29	
**MCH**	pg	30,01	29,55	30,09	29,79	30,26	26,50	26,63	26,77	26,76	26,72	
**MCHC**	g/dl	33,87	33,51	33,99	33,46	33,98	33,02	33,08	33,48	33,30	33,27	
**EVB**	%	12,99	13,11	13,18	12,78	13,26	14,41	13,87	13,89	13,77	13,83	
**EVB-SD**	fL	39,38	39,81	40,25	38,94	40,69	40,69	38,50	38,94	38,50	38,94	
**THR**	10^3/µl	195,80	88,70	95,90	68,30	82,60	97,50	20,00	6,60	28,90	6,80	
**MTV**	fL	9,10	7,16	6,88	7,56	7,43	8,06	5,62	9,16	6,47	9,45	
**freies Hb**		0,02	0,03	0,03	0,03	0,02	0,04	0,02	0,03	0,04	0,02	1,74

		MW					Stabw				
		**blood contr**	**Ti**	**316L**	**CoCrMo**	**P2000**	**blood contr**	**Ti**	**316L**	**CoCrMo**	**P2000**
**Quick**	s	12,20	11,15	11,30	11,35	11,10	0,14	0,35	0,42	0,49	0,28
	%	88,00	102,50	100,00	99,00	103,00	0,00	3,54	4,24	5,66	2,83
	INR	1,09	0,99	1,00	1,01	0,98	0,01	0,02	0,03	0,04	0,01
**PTT**	s	28,20	28,70	28,90	28,55	28,00	4,38	3,39	4,95	4,31	3,39
**Fib-C**	s	16,05	14,35	14,55	14,65	14,15	0,21	0,21	0,64	0,92	0,92
	mg/dl	195,50	229,50	225,50	223,50	235,50	0,71	12,02	20,51	26,16	28,99
**Ddim**	µg/ml	0,18	0,20	0,23	0,25	0,24	0,04	0,08	0,03	0,03	0,00
	mAbs	1,73	2,34	2,91	3,54	3,22	1,08	2,09	0,71	0,52	0,08
**PTZ5**	s	15,75	12,35	12,50	12,60	12,20	0,78	0,07	0,42	0,42	0,42
**LEU**	10^3/µl	4,63	4,52	4,65	4,50	4,62	0,11	0,20	0,02	0,22	0,21
**ERY**	10^6/µl	4,67	4,71	4,62	4,66	4,67	0,90	0,94	0,80	0,83	0,91
**HGB**	g/dl	13,09	13,14	13,05	13,08	13,21	1,38	1,68	1,20	1,35	1,42
**HKT**	%	39,17	39,48	38,69	39,18	39,31	4,82	5,41	3,95	4,16	4,80
**MCV**	fL	84,42	84,34	84,24	84,69	84,67	5,92	5,44	6,05	6,14	6,19
**MCH**	pg	28,26	28,09	28,43	28,28	28,49	2,48	2,06	2,35	2,14	2,50
**MCHC**	g/dl	33,45	33,30	33,74	33,38	33,63	0,60	0,30	0,36	0,11	0,50
**EVB**	%	13,70	13,49	13,54	13,28	13,55	1,00	0,54	0,50	0,70	0,40
**EVB-SD**	fL	40,04	39,16	39,60	38,72	39,82	0,93	0,93	0,93	0,31	1,24
**THR**	10^3/µl	146,65	54,35	51,25	48,60	44,70	69,51	48,58	63,14	27,86	53,60
**MTV**	fL	8,58	6,39	8,02	7,02	8,44	0,74	1,09	1,61	0,77	1,43
**freies Hb**		0,03	0,03	0,03	0,04	0,02	0,01	0,01	0,00	0,01	0,00

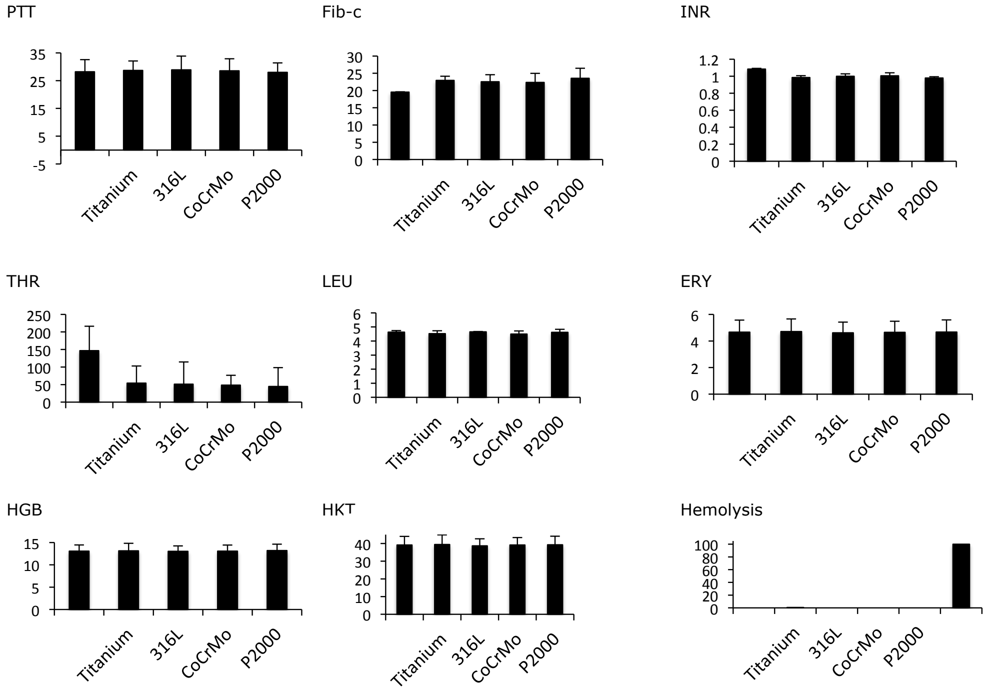

Platelets

Ti			316L			CoCrMo			P2000		
#1	#2	#3	#1	#2	#3	#1	#2	#3	#1	#2	#3
120	55	210	210	245	195	170	220	165	220	300	105
85	105	150	160	280	40	150	280	70	210	150	85
37	180	35	225	270	60	130	210	225	160	210	145
340	310	130	105	230	50	220	315	80	205	200	220
145	235	145	125	370	225	210	200	100	320	240	180

Titaniu	316L	CoCrMo	P2000
152	186	183	197
91	95	70	64

	Titaniu	316L	CoCrMo	P2000
MW	100	122	120	129
Stabw	60	62	46	42

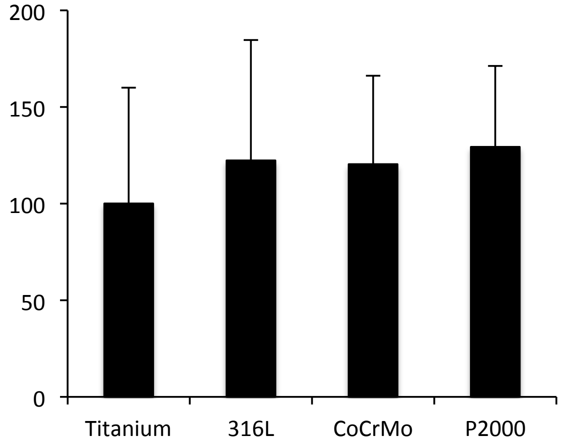

Hardness

H GPa				
**Messung**	**Titanium**	**316L**	**P2000**	**CoCrMo**
1	3,8	3,8	5,1	6,8
2	4,5	4,2	5,7	6,7
3	3,8	4,3	5,7	7,6
4	3,9	4,1	5,7	7,4
5	3,8	3,9	5,8	7,2
6	4,1	4,2	5,6	7,3
7	4,2	4,3	5,9	8,2
8	3,8	4,3	5,6	8
9	4,2	4,5	5,6	8,5
10	4,2	5,2	5,1	7,6
MW	4,03	4,28	5,58	7,53
Stdabw	0,25	0,38	0,27	0,58

	Titanium	316L	CoCrMo	P2000
MW	4,03	4,28	7,53	5,58
Stdabw	0,25	0,38	0,58	0,27

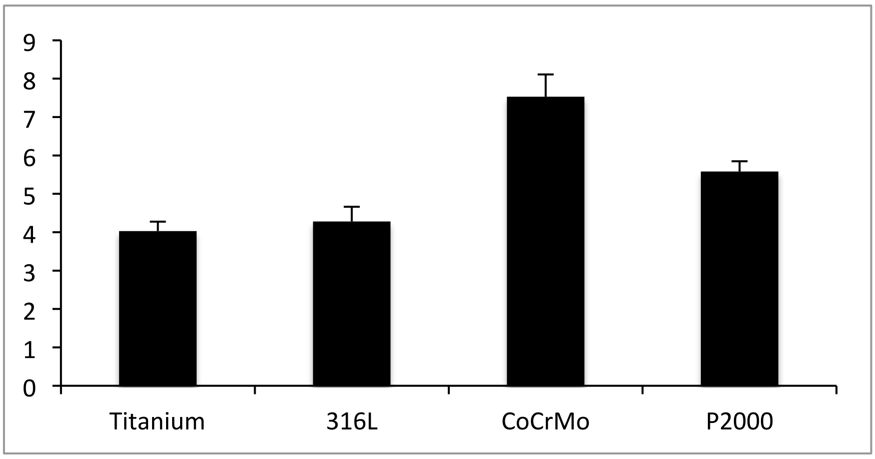

Supplement: S1 Dataset — (PDF) [file pone.0214384.s002.pdf]
